# Localized Intrathecal Delivery of Mesenchymal Stromal Cells Conditioned Medium Improves Functional Recovery in a Rat Model of Spinal Cord Injury

**DOI:** 10.3390/ijms19030870

**Published:** 2018-03-15

**Authors:** Dasa Cizkova, Veronika Cubinkova, Tomas Smolek, Adriana-Natalia Murgoci, Jan Danko, Katarina Vdoviakova, Filip Humenik, Milan Cizek, Jusal Quanico, Isabelle Fournier, Michel Salzet

**Affiliations:** 1 Institute of Neuroimmunology, Slovak Academy of Sciences, Dúbravská cesta 9, 845 10 Bratislava, Slovakia; veronika.cubinkova@savba.sk (V.C.); tomas.smolek@savba.sk (T.S.); adriana.murgoci@savba.sk (A.-N.M.); 2 Department of Anatomy, Histology and Physiology, University of Veterinary Medicine and Pharmacy in Košice, Komenského 73, 041 81 Košice, Slovakia; jan.danko@uvlf.sk (J.D.); katarina.vdoviakova@uvlf.sk (K.V.); filip.humenik@uvlf.sk (F.H.); 3 Université de Lille, Inserm, U-1192—Laboratoire Protéomique, Réponse Inflammatoire et Spectrométrie de Masse-PRISM, F-59000 Lille, France; Jusal.Quanico@univ-lille1.fr (J.Q.); isabelle.fournier@univ-lille1.fr (I.F.); michel.salzet@univ-lille1.fr (M.S.); 4 Department of Epizootology and Parasitology, University of Veterinary Medicine and Pharmacy in Košice, Komenského 73, 041 81 Košice, Slovakia; milan.cizek@uvlf.sk

**Keywords:** spinal cord injury, inflammation, regeneration, stem cells-derived conditioned media

## Abstract

It was recently shown that the conditioned medium (CM) of mesenchymal stem cells can enhance viability of neural and glial cell populations. In the present study, we have investigated a cell-free approach via CM from rat bone marrow stromal cells (MScCM) applied intrathecally (IT) for spinal cord injury (SCI) recovery in adult rats. Functional in vitro test on dorsal root ganglion (DRG) primary cultures confirmed biological properties of collected MScCM for production of neurosphere-like structures and axon outgrowth. Afterwards, rats underwent SCI and were treated with IT delivery of MScCM or vehicle at postsurgical Days 1, 5, 9, and 13, and left to survive 10 weeks. Rats that received MScCM showed significantly higher motor function recovery, increase in spared spinal cord tissue, enhanced GAP-43 expression and attenuated inflammation in comparison with vehicle-treated rats. Spared tissue around the lesion site was infiltrated with GAP-43-labeled axons at four weeks that gradually decreased at 10 weeks. Finally, a cytokine array performed on spinal cord extracts after MScCM treatment revealed decreased levels of IL-2, IL-6 and TNFα when compared to vehicle group. In conclusion, our results suggest that molecular cocktail found in MScCM is favorable for final neuroregeneration after SCI.

## 1. Introduction

Spinal cord injury (SCI) is one of the major traumas of the central nervous system (CNS) leading to devastating neurological outcomes [1,2]. Unfortunately, there are few approved treatments available for stabilizing this condition and none for effective treatment [3,4,5]. Both animal and human studies provide evidence of multiple factors such as inflammation, reactive gliosis, axonal demyelination, neuronal death and cysts formation being the driving forces behind the secondary injury processes of SCI [6,7,8]. Furthermore, the regenerative capacity of the adult CNS tissue at the injury site is significantly limited particularly due to the accumulation of inhibitory molecules and loss of trophic factors [9,10,11]. Therefore, the regeneration of injured nerve tissue is often a temporary process, which can occur only at a short distance and during limited period, thus representing a challenge for the development of new therapeutic strategies [12]. 

Indeed, in recent years, convincing immunomodulatory and neurotrophic properties have been ascribed to mesenchymal stem cells (MSc) derived from the bone marrow showing partial beneficial impact on locomotor function [13,14,15,16]. However, their true therapeutic properties rely on released secretomes comprising a soluble fraction of proteins, growth factors, cytokines and a vesicular fraction composed by microvesicles and exosomes [17]. Considering these facts, it seems that the secretomes of the MSCs appear to be of greater benefit on tissue regeneration and repair than the stem cells themselves [18]. Recently, essential attention has been paid to MSc-derived exosomes that are involved in the transmission of proteins and genetic material (e.g., mi RNA) to neighboring cells [19]. Using mass spectrometry, we have analyzed under in vitro conditions bioactive substances produced by rat MSc. The results confirmed that rat MScCM contains growth and migratory factors for neurons, factors for osteogenic differentiation and immunomodulatory factors [14]. Functional tests using chemotactic analysis in vitro confirmed that MScCM reduces migration activity in LPS-activated BV2 cells and primary microglia and stimulated nerve outgrowth in DRGs explants [14]. 

Furthermore, we have confirmed therapeutic efficacy of intrathecal administration of PKH67-labelled MSc by improving motor function of the hind paws after SCI [20]. Histological, morphometric and stereological analyses confirmed that transplanted MSc survived, migrated and were incorporated into the lesion site, where we have seen an increased number of growing nerve fibers [20]. Our results indicate that intrathecal administration of stem cells is a non-invasive method for local delivery of optimal dose leading to overall recovery improvement. Similarly, our previous work with systemic administration of MSc showed a therapeutic effect [21]. Furthermore, a cell-based strategy combined with bio-materials may be prospective in terms of proposing more effective approaches [22,23]. Indeed, transplanted MSc alone or seeded into smart scaffolds may fulfill the role of “pharmacies” which are providing paracrine factors mediating therapeutic benefits on demand of the host tissue [24].

As opposed to cell therapies, the local delivery of secretomes gets to the forefront of interest for possible treatment of CNS injuries. These secretomes must supply the injury site with bioactive molecules for a sufficient time to achieve optimized clinical outcomes. Therefore, frequent delivery during longer periods from about two weeks should be considered.

Supporting this concept, here we determine whether localized, intrathecal delivery of the MScCM affects tissue sparing, axonal regrowth, immune response and functional outcome after SCI. The designed CM treatment represents a promising alternative to traditional stem cell therapy for the treatment of acute spinal cord injury.

## 2. Results

### 2.1. Functionality of CM

#### 2.1.1. High-Density Plating Neurosphere-Like Structures Formation 

**Figure 1:**
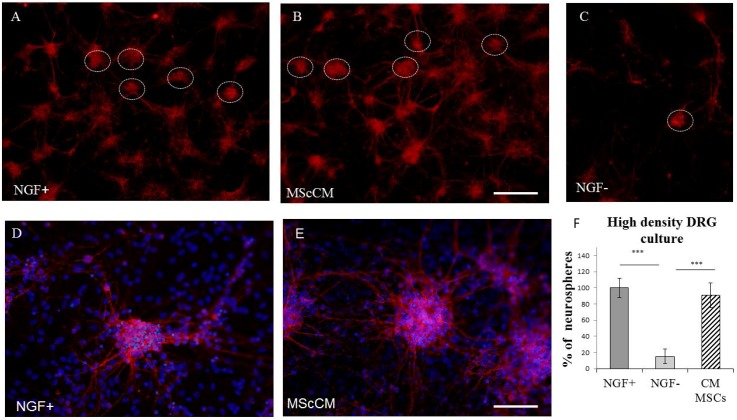
Formation of neurosphere-like structures (shown in white circles) from TUJ1 positive PCDRGs plated in high-density, supplemented: with NGF (**A**,**D**); with MScCM (**B**,**E**); and without NGF (**C**). Quantification of neurosphere-like structures (**F**). *** *p* < 0.001 indicate significant differences between groups. Scale bars: (**A**–**C**) = 400 μm; and (**D**,**E**) = 100 μm.

Primary cultures of DRGs (PCDRGs) plated at a high density (800–1000 cells/mm^2^) continuously multiplied and formed neurosphere-like structures during one week. Immunohistochemistry using TUJ1 confirmed that these spheres were interconnected via extended neurites and formed almost confluent neuronal networks (Figure 1A,B,E). This pattern was observed when PCDRGs were cultured in medium supplemented with nerve growth factor (NGF), and MScCM (Figure 1A,B,D,E), but not when they were cultured in the basic medium (lacking trophic molecules) (Figure 1C). Quantification of neurosphere-like structures did not show differences between PCDRGs cultures with NGF and MScCM supplementation (Figure 1F). 

#### 2.1.2. Low-Density Plating 

**Figure 2:**
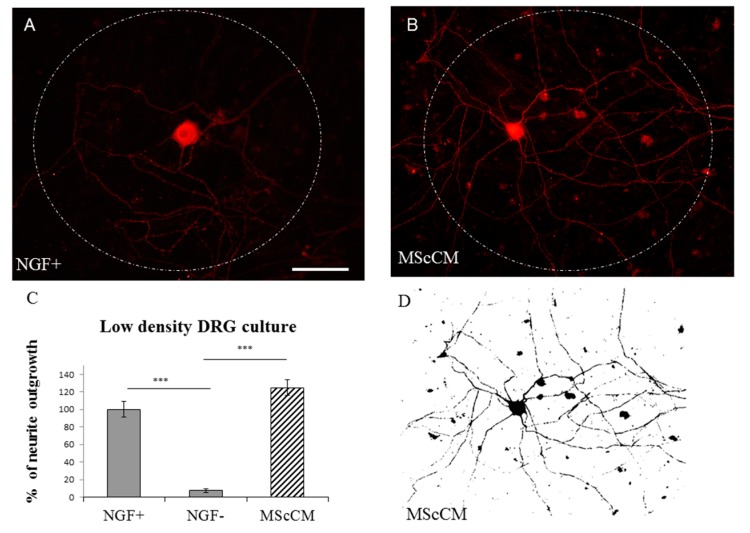
Neurite outgrowth of low-density plated PCDRGs, TUJ1 positive supplemented with: NGF (**A**); and MScCM (**B**). Quantification of neurite outgrowth. *** *p* < 0.001 indicates significant differences between groups (**C**). The image indicates TUJ1 stained cell with neurites after selecting the area to be analyzed and thresholding the number of pixels covered by the extending neuritis (Image J) (**D**). *** *p* < 0.001. Scale bars: (**A**,**B**,**D**) 50 μm.

For quantification of delicate neurite outgrowth, we have used low-density plating (30–50 cells/mm^2^). Under this condition, the surface area outside the cell body that was covered with delicate neurites was determined (Figure 2D). This showed that enhanced TUJ1 positive neurite outgrowth from cell body was stimulated with NGF+ or MScCM supplemented culture media (Figure 2A,B). Conversely, almost no outgrowth from sensory neurons occurred after incubation without NGF (Figure 2C). The mean percent of neurite outgrowth for each group was: 100% ± 9.1 = NGF+; 7.07% ± 2.28 = NGF−; 124.97% ± 9.2 = MScCM; *** *p* < 0.001. 

### 2.2. Locomotor Function Recovery

**Figure 3:**
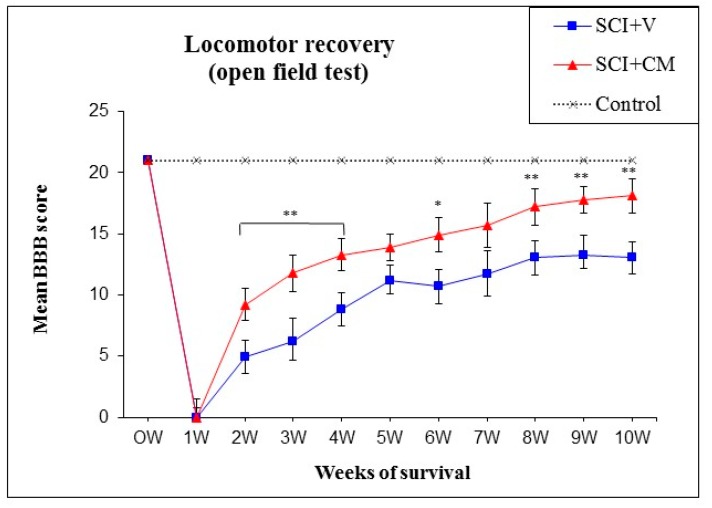
Functional recovery of hindlimb motor function following spinal cord injury (SCI) treated with MScCM (SCI + CM, *n* = 6) and vehicle (SCI + V, *n* = 4). * *p* < 0.05, ** *p* < 0.01 indicate significant differences between groups during ten weeks, respectively.

During the initial days post-injury, contusion caused hindlimb paralysis with slight movement in one or two joints in all experimental groups. On following days, the animals in the SCI + CM treatment groups showed a gradual recovery of hindlimb locomotion up to ten weeks after SCI; greater than the recovery in the SCI + V groups, where limited recovery of motor function was noted. The significant locomotor improvement between injured groups without treatment (SCI + V) and treated group (SCI + CM) was detected at two weeks, three weeks, four weeks (** *p* < 0.01), six weeks (* *p* < 0.05), 8–10 weeks (** *p* < 0.01) post-injury with the following BBB scores: 9.2 ± 1.3/two weeks, 11.8 ± 1.5/three weeks, 13.3 ± 1.4/four weeks, 13.9 ± 1.1/five weeks, 14.9 ± 1.4/six weeks, 15.7 ± 1.8/seven weeks, 17.2 ± 1.5/eight weeks, 17.8 ± 1.1/nine weeks, 18.1 ± 1.4/ten weeks (SCI + MScCM); and 4.9 ± 1.4/two weeks, 6.2 ± 1.9/three weeks, 8.8 ± 1.4/four weeks, 11.2 ± 1.2/five weeks, 10.7 ± 1.4/six weeks, 11.7 ± 1.9/seven weeks, 13.1 ± 1.3/eight weeks, 13.3 ± 1.6/nine weeks, 13.1 ± 1.2/ten weeks (SCI + V) (Figure 3).

### 2.3. Spared Spinal Cord Tissue

**Figure 4:**
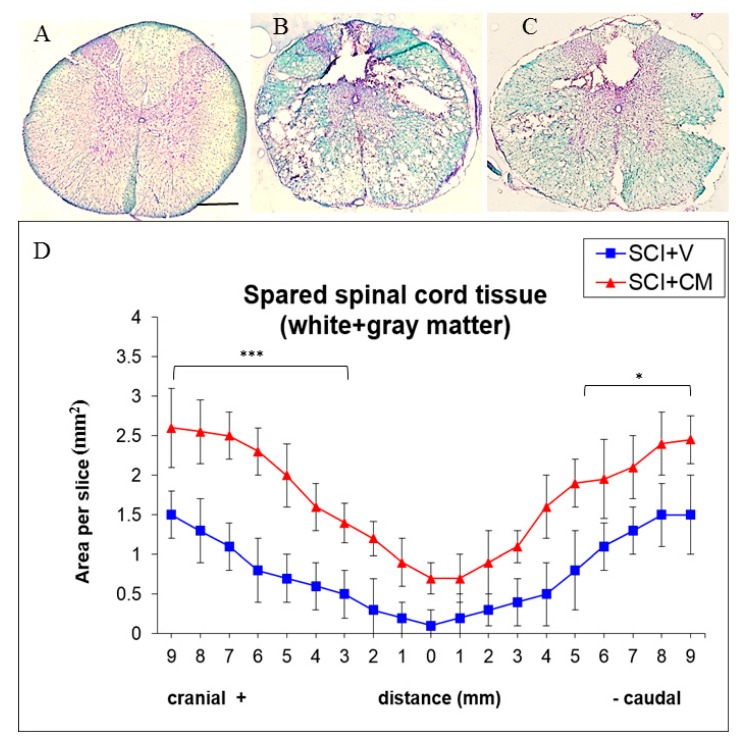
Representative cross section of spinal cord, Th8 stained with luxol fast blue of: control (**A**); SCI treated with vehicle (SCI + V) (**B**); and treated with MScCM (SCI + CM) (**C**). Quantitative stereological analyses of tissue fenestration in 1.8 cm (0.9+/−) segment (**D**). Note, there are significant differences of spared tissue (gray and white matter) between SCI + V (blue line) and SCI + CM (red line) treatment in most cranial and caudal sections (*** *p* < 0.001, * *p* < 0.05). Scale bars (**A**–**C**) = 500 μm.

Histological assessment of spared tissue (gray and white matter together) was done with luxol fast blue/cresyl violet staining on coronal spinal cord sections taken from rats processed to SCI + V and SCI + CM treatment (Figure 4B,C). Staining showed an elevated amount of spared tissue in SCI + CM group (Figure 4C) compared to SCI + V (Figure 4B) at ten weeks post-surgery (Figure 4D). Quantitative stereological analyses of tissue fenestration in 1.8 cm (0.9+/−) segment revealed significant differences among samples studied in most +cranial 9,8,7,6,5,4,3 mm (*** *p* < 0.01) and −caudal sections 5,6,7,8,9 mm (* *p* < 0.05); CM treated group: +cranial 9,8,7,6,5 mm = 2.6, 2.5, 2.5, 2.3, 2.0/±0.1–3 mm; −caudal 5,6,7,8,9 mm = 1.9, 1.9, 2.1, 2.4, 2.4, 2.1/±0.2–5 mm. Compared to the vehicle treated group; +cranial 9,8,7,6,5 mm = 1.5, 1.3, 1.1, 0.8, 0.7/±0.2–0.5 mm; −caudal 5,6,7,8,9 mm = 0.8, 1.1, 1.3, 1.5, 1.5/±0.2–5 mm) (*** *p* < 0.01, * *p* < 0.05) (Figure 4).

### 2.4. Neurite Sprouting

**Figure 5:**
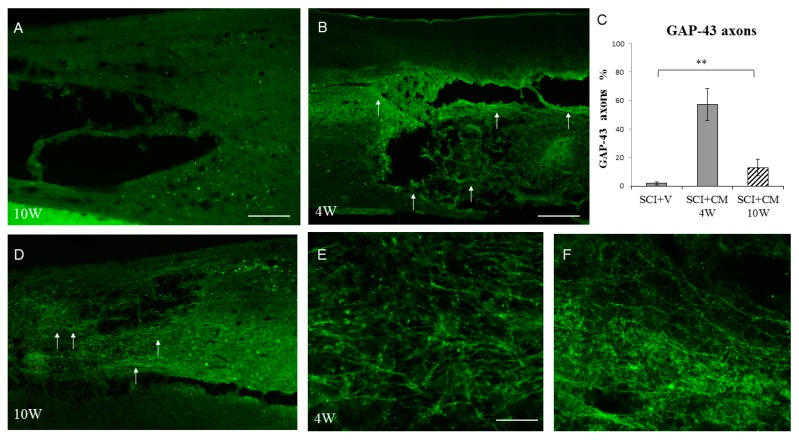
Immunohistochemistry revealing the presence of GAP-43-positive axons located in: lesion (**B**, arrows indicate highest positivity), the rostral (**E**), and caudal segments (**F**) in SCI + CM after four weeks. Note the significant decrease of GAP-43 fibers at ten weeks (**D**, arrows indicate occasional positive axons), and no expression in SCI + V group (**A**). Quantification of GAP-43-positive axons between groups (**C**) ** *p* < 0.01. Scale bars: (**A**,**B**,**D**) 400 μm; and (**E**,**F**) 150 μm.

CM-mediated treatment promoted growth of GAP-43-labeled axon fibers that were infiltrating the lesion site and adjacent segments (Figure 5B,E,F). The immunohistochemistry analysis revealed the presence of GAP-43-positive axons located in the lesion (Figure 5B), the rostral (Figure 5E), as well as in the caudal segments (Figure 5F). Furthermore, the increased density of axon fibers forming a multiplicity sprouting and enhanced regrowth through the cavity showed significant differences at four weeks (57.2 ± 11.2% axons) and ten weeks (12.9 ± 5.7% axons) when compared with the vehicle group (SCI + V) at four weeks (2.2 ± 0.7% axons) (Figure 5C). The majority of GAP-43-labeled axons were seen in gray matter of MScCM treated group at four weeks (Figure 5B,E,F), and significantly decreased at ten weeks (Figure 5C,D). Immunocytochemical analysis of SCI + V sections revealed no or very low expression of GAP-43 fibers (Figure 5A); ** *p* < 0.01. 

### 2.5. Cytokines Profile

**Figure 6:**
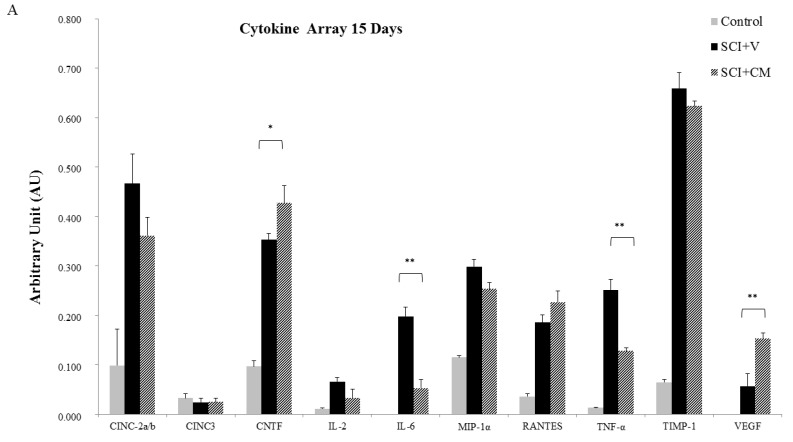

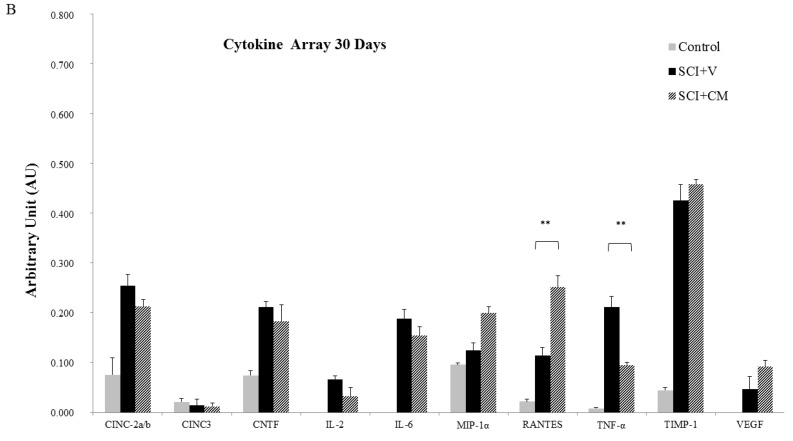
Chemokines and cytokines array after SCI in conditioned media from following groups: Control, SCI + V, SCI + CM. Comparison of cytokines and chemokines secretion at: 15 days (**A**); and 30 days (**B**) in studied groups. Bar diagrams represent the ratio of the spot mean pixel densities/reference point pixel densities. Statistical significance indicated by * *p* < 0.05, ** *p* < 0.01.

Cytokine arrays confirmed the time-dependent synthesis of chemokines and cytokines after SCI. Compared with control, CINC-2a/b, IL2, IL6, MIP-1α, Rantes, CXCL3, TNFα, TIMP-1 and VEGF were significantly overexpressed 15 days after SCI (Figure 6A). At the same interval, treatment with CM significantly decreased IL6 and TNFα, while CNTF and VEGF continue to increase (Figure 6A). Cytokine and chemokine profile between SCI + V and SCI + CM has gradually evened out at ten weeks, although the levels were still higher compared to the control (Figure 6B). Taken together, these data showed that the cytokine pattern changes in time course between SCI + V and SCI + CM treatment and CM may temporarily decrease inflammatory response.

## 3. Discussion

We have previously reported that IT delivered MSc migrated and incorporated into the central lesion leading to higher motor function recovery [20]. Thus, in the context of these findings, the main objective of the present study was to test whether conditioned medium derived from MSc may have similar beneficial properties for the recovery of the damaged spinal cord as cell-based treatments. First, we confirmed the functionality of MScCM biological activity in vitro on neonatal DRGs primary cultures. Supplementation of primary sensory neurons with CM stimulated formation of neurospheres and neurite outgrowth similarly as was seen after administration of NGF. These in vitro results confirm the functionality of neurotrophic and mitotic molecules released from MSc that were identified by MS/MS in our previous study [14]. 

Based on these results, we developed a regimen for IT CM delivery. We had to consider the optimal dose and therapeutic window to ensure a continuous supply of trophic molecules to the site of injury. Since we were delivering stem cell products that have limited biological activity, we needed to continually supply the injury site with sufficient amount of trophic molecules. Therefore, we decided to administer CM at 4-day intervals from 1 day to 13 days after injury. Four-day interval was selected due to our previous in vitro data, which showed that the supplementation of neural progenitors with trophic factors (EGF, FGF) every 3–4 days is sufficient to maintain their biological activity and to stimulate their neurogenic behavior (growth and proliferation of neurospheres). Furthermore, the pro–regenerative and inflammatory processes are initiated within the first week after SCI, which outline this period as a potential therapeutic window for treatment intervention [25,26]. 

In context with this strategy, our behavioral tests confirmed a progressive improvement of the motor function in the SCI + CM treated group compared to the untreated one (SCI + V). We noticed the most significant changes during the first 2–3 weeks and then from 8–10 weeks, although in the vehicle group there was small number of animals that survived up to 10 weeks. Therefore, for future studies we need to work with larger groups in order to support behavioral data. This beneficial outcome correlates with the spared white and gray matter, which was approximately 30% higher in the SCI + CM group. Furthermore, conditioned medium-induced sparing of spinal cord tissue should logically correlate with enhanced regenerative processes. For this reason, we have studied the expression of GAP-43-positive axons, which correspond to newly outgrowing axons with the capacity of re-building the neural connections after CNS damage. The regeneration in adult tissue can be initiated only for a few days when GAP-43-positive fibers are able to grow over short distances [27,28]. However, stimulation of GAP-43-positive axons by exogenous delivery of stem cells or by limiting the inhibitory environment can significantly increase the amount of GAP-43-positive fibers over a longer period [21,28]. This fact may significantly prolong the regeneration process with overcoming the nerve fibers over the formation of a non-functional glial scar leading to improved outcome [10,29]. 

Another important factor influencing regeneration that needs to be considered is the severe inflammatory response after SCI. In the CM treated group, we have experienced a significant decrease in IL6 and TNFα and an increase in the trophic factors of CNTF and VEGF, which were gradually reduced with time to the levels observed in the untreated group. This points to the fact that MScCM is capable of temporarily modulating limited immune response as we previously demonstrated on microglia cells [25]. This is due to MSc secreted factors. In fact, MScCM is known to contain anti-inflammatory factors (TGFβ (1,2,3), osteopontin) which impacts activated microglia [30] and infiltrated macrophages. However, to achieve stronger immunomodulation we need inter-cellular response, rather than their products. This was confirmed in another study showing that IT delivery of MSc reduced inflammation via suppression of a broader scope of cytokines (TNFα, IL-4, IL-1β, IL-2, IL-6 and IL-12) and increased the levels of MIP-1α and RANTES [15]. Furthermore, in a recent study, the authors showed that MSc-derived exosomes specifically target M2-type macrophages at the site of SCI [31]. This supports the idea that extracellular vesicles, released by MSc, may mediate the therapeutic effects towards immune response modulation [32].

Indeed, these results positioned the MScCM treatment on the level of cell based therapy, where similar beneficial outcomes were observed [15,20]. The next step of this project will be isolation of the microvesicles (exosomes) from the MScCM as novel therapeutic agents as we recently demonstrated for microglia cells [33].

## 4. Materials and Methods 

### 4.1. MSc Culture and Conditioned Media (MScCM) Collection

MScs were isolated from the bone marrow of four 10-week old male Wistar rats (300 g), collected from the long bones (femur and tibia) [14,20]. The bone marrow was dissected into small pieces, gently homogenized, and filtered (70 µm) to remove bone fragments and centrifuged. The cell pellet was re-suspended in 1 mL of alpha-Minimum essential media (MEM), the pooled cells were counted, and their viability was assessed using the trypan blue dye exclusion method. MScs were subsequently resuspended in culture medium composed of alpha-MEM supplemented with 10% of fetal calf serum (FCS; GIBCO Laboratories, Grand Island, NY, USA) and antibiotics (10,000 units/mL penicillin, 10,000 mg/mL streptomycin, and 25 mg/mL amphotericin B; Invitrogen, Carlsbad, CA, USA), and plated at a density of 30,000 cells/cm^2^ in uncoated tissue culture flasks, as these cells readily adhere to the plastic. The cells were incubated in a humidified atmosphere with 5% CO_2_ at 37 °C. Non-adherent cells were removed after 3–4 days by medium change and the remaining cells were fed twice per week. When the cultures reached 80% of confluence, the MScs were passaged with 0.25% trypsin/0.53 mM Ethylene diamine tetraacetic acid (EDTA; GIBCO Laboratories, Paisley, UK), centrifuged, and re-plated at a density of 5000 cells/cm^2^. The MScs were propagated for three passages and then characterized as previously described [14]. 

Cells at passage 3 cultured in Dulbecco’s modification of Eagle’s medium (DMEM, Sigma Aldrich, Saint Louis, MO, USA) with low glucose and without fetal bovine serum were incubated in a humidified atmosphere with 5% CO_2_ at 37 °C for 24 h and used for MScCM collection, using a similar protocol as in the previous study [14]. 

### 4.2. Primary Cultures of Dorsal Root Ganglion Neurons (PCDRGs) 

Dorsal root ganglia (DRGs) located in cervical to sacral spinal levels were dissected from newborn (postnatal day 1 (P1), *n* = 50) Wistar rats following decapitation. Under sterile conditions and using a stereomicroscope the DRGs were cut into smaller pieces and incubated at 37 °C in 3 mL collagenase dissolved in a dissociation solution consisting of HBSS supplemented with collagenase (concentration of 500 UI/mL; collagenase from Clostridium histolyticum C5138, Sigma Aldrich, Saint Louis, MO, USA), hyaluronidase (concentration of 150 UI/mL; hyaluronidase Type IV, Sigma Aldrich-Chemie GmbH, Buchs, Switzerland). The digestion process was stopped by adding an equal volume of HBSS, and the suspension was filtered through a stainless mesh sieve (pore size 100 μm), centrifuged for 5 min (1000× *g*) and the supernatant was discarded. Cells were divided into 3 groups: (1) NGF+ group, grown in DMEM F12 medium with 1% fetal bovine serum and nerve growth factor (2.5S NGF; 50 ng/mL); (2) NGF− group, grown in DMEM F12 medium with 1% fetal bovine serum; and (3) MScCM group, grown in MScCM in DMEM F12 medium and 1% fetal bovine serum (ratio 3:1). All three groups were supplemented with 1% penicillin and streptomycin and allowed to adhere overnight onto poly-dl-ornithine (500 μg/mL) and laminin (10 ng/mL) coated glass coverslips in 12-well tissue culture plates (Costar, Corning, New York, NY, USA) at 37 °C for 7days in vitro (7DIV). For all PCDRGs, we have used standard IHC procedures to visualize neurite outgrowth. PCDRGs were incubated in mouse anti-Neuron-specific class III beta-tubulin (TUJ1) (1:200; Merck, Darmstadt, Germany; Molecular Probes, Eugene, OR, USA) for 24 h at 4 °C. Afterwards, they were washed in 0.1 M PBS and incubated for 2 h with goat anti-mouse IgG (AlexaFlour 488, Molecular Probes, Eugene, OR, USA). For nuclear staining, we used 4-6-diaminidino-2-phenylindol (DAPI, 1:200) and finally the slides were coverslipped with Vectashield mounting medium (Vector Laboratories, Inc., Burlingame, CA, USA). Digitized images of PCDRGs/per treatment (*n* = 6) were captured and saved with NIS-Elements Imaging Software (Nikon, Prague, Czech Republic). Neurite outgrowth was analyzed at identical sampling fields, for each CM experiment by ImageJ software according to the above-mentioned method applied for primary antibodies quantification, according to our previous study [14].

### 4.3. Intrathecal Implant Procedure

Intrathecal (IT) catheter implantation was performed according to our previously published procedure [20]. The rats were anesthetized with 1.5–2% halothane, and following a loss of responsiveness, the head was fixed in a stereotaxic head holder. The atlanto-occipital membrane was blotted dry to visualize the entire area and a 3 to 4 mm incision was made through the dural midline using a 30-gauge needle. The PE-5 end of the IT catheter was inserted into the IT space and advancing the tip of the catheter along the spinal cord for a distance up to 3.5 cm. The opposite PE-10 catheter end was externalized on the forehead and the end was plugged with 4–5 mm of stainless steel 28-gauge wire. The overlying muscles and skin were sutured using a silk suture to fix the catheter in place. All implanted IT catheters were flushed with 15.0 μL of sterile saline for 10 min to test their transition, and in the case of an obstruction, the catheter was repositioned or exchanged. After recovery, each rat was evaluated for additional limb dysfunction, spinal asymmetry, pain or adverse surgical effects, and sacrificed immediately if any of these were observed.

### 4.4. Spinal Cord Trauma

All experimental procedures were approved by the institutional ethical committee for animal research, and were in accordance with the Slovak Law for Animal protection (No. 39/2007, 12 December 2006). SCI was induced using the modified balloon compression technique in adult male Wistar rats (*n* = 24) weighing between 300 and 320 g, according to our previous study [21]. The SCI was induced by balloon-technique in adult male rats. Surgery was performed under anesthesia with 1.5–2% halothane in air delivered through a face mask. Using a dental drill, a small hole was made at Th8–9 level and a 3-French Fogarty catheter was inserted epidurally and the balloon was inflated with 12.5 μL of saline for 5 min. SCI was also associated with damage of the autonomic nervous system, which includes regulatory functions of urinary bladder. For this reason, manual bladder expression was required (twice a day) until bladder reflex was restored. No antibiotic treatment was used.

### 4.5. IT Delivery of MSCs Conditioned Medium (MScCM)

All SCI rats were randomly divided into the following groups: (1) MSc conditioned medium (CM) group receiving four daily injections of 30 µL MScCM (SCI + CM) at 1, 5, 9 and 13 days; or (2) a vehicle group (SCI + V) receiving 30 µL of DMEM per rat at the same intervals. A maximum dose of 30 µL CM was flushed with 5 µL of saline over 30 min through a calibrated PE10 catheter (calibrated by 5-µL increments up to 35 µL), which was connected to a 500-µL Hamilton syringe attached to a mechanical mini-pump. This procedure was performed in each rat of the SCI + CM and vehicle groups. Implantation of the IT catheter was performed on the same day as IT delivery, at 1 day after SCI. Animals were intracardially perfused at 15 days and four weeks (*n* = 8/IT delivery CM, *n* = 6/IT delivery vehicle) and at ten weeks after SCI (*n* = 6/IT delivery CM, *n* = 4/IT delivery vehicle).

### 4.6. Behavioral Testing of Motor Function (BBB Scoring)

Animals were evaluated before surgery and once a week after surgery for 10 weeks. Each rat was tested for 5 min by two blinded examiners. The motor performance was assessed using the Basso–Beattie–Bresnahan (BBB) 21-point open field locomotor scale [16]. BBB scores, which categorize combinations of rat hindlimb movements, trunk position and stability, stepping, coordination, paw placement, toe clearance and tail position, were analyzed. In this evaluation, 0 represents no locomotion and 21 represents normal motor function.

### 4.7. Cytokines Profile 

Cytokines profile of CM derived from control, SCI + V, SCI + CM after 15 days and 30 days for the segment R1 was performed by using a Rat Cytokine Array Panel A according to the manufacturer’s instructions (R&D Systems, Inc., Abingdon, UK). Conditioned media from rostral segments from spinal cord tissue (all experimental groups and control) were obtained according to our previous study and afterwards processed for Cytokine profile detection [25]. We have used a similar procedure as in our previous study [26]. Briefly, the array membranes were first incubated in the blocking buffer for 1 h. Afterwards 200 µL of CM were mixed with the detection antibody mixture and incubated for 1 h at room temperature. After removing the blocking buffer, the sample/antibody mixture was added to array membranes and incubated overnight at 4 °C. Next day, the membranes were washed with the buffer and then incubated with Streptavidin-HRP solution for 30 min at room temperature. The membranes were finally washed with buffer 4 times and the bound antibodies were detected by chemoluminescence using a chemireagent mix. The membranes were quantified by densitometry using ImageJ software. Background staining and spot size were analyzed as recommended by the manufacturer. Normalization was done with control expression.

### 4.8. Morphometric Analysis 

To analyze the amount of spared spinal cord tissue, we have performed modified luxol fast blue histological staining according to our procedure [22]. A 1.8 cm-long segment of the spinal cord was dissected between 0.9 cm cranial and 0.9 cm caudal to the injury epicenter. Spinal cords were transversally cut at 1-mm intervals along the cranio-caudal axis from the lesion center and stained with cresyl violet and luxol-fast blue. Five sections were selected at 1-mm intervals and whole images of the spinal cord were taken with Leica plus microscope digital camera (Microsystems, Mannheim, Germany). We calculated the ratio between the measured injured area and the entire area of the spinal cord slice (white + gray matter) on serial transversal sections (+9 mm; 0; −9 mm) by Image J software (Wayne Rasband, National Institutes of Health, Bethesda, MD, USA). Mean number of spared tissue of evaluated groups was expressed in mm^2^. 

### 4.9. Immunohistochemistry and Quantification Analysis

Experimental and control rats were deeply anesthetized with a ketamine-xylazine cocktail (15 mg/kg of xylazine and 150 mg/kg of ketamine), and transcardially perfused with 0.1 M PBS, followed by 2% paraformaldehyde in PBS. The spinal cord and externalized IT catheter was removed, post-fixed in the same fixative solution overnight at 4 °C, and embedded in the gelatin albumin substrate in 2% paraformaldehyde in PBS. Frozen spinal cord sections were cut from a 1.5-cm-long spinal cord segment positioned on the injury epicenter, embedded in embedding medium, dissected into three 0.5-cm-thick blocks (rostral, epicenter, and caudal), and stored at −20 °C. Sections 10-µm thick were then serially cut from the epicenter and mounted directly onto slides. From the rostral and caudal regions, 40-µm-thick cryostat (Leica) sections were cut and collected in 24-well plates with 0.1-M PBS containing 0.1% sodium azide. Sections were taken at 200 µm intervals and 20 sections per block were obtained. Ten sections per block were stained with hematoxylin and eosin (H&E) to assess tissue morphology and determine the injury epicenter. For immunohistochemistry, free-floating sections (40 μm) were immersed in PBS (0.1 M; pH 7.4) containing 10% normal goat serum (NGS), 0.2% Triton X-100 for 2 h at room temperature to block non-specific protein activity. This was followed by overnight incubation at 4 °C with primary antibodies: mouse anti-GAP-43 (1:1000, Sigma, Saint Louis, MO, USA) for 24 h. Afterwards sections were washed in 0.1 M PBS and incubated with secondary fluorescent antibodies goat anti-mouse conjugated fluorescein isothiocyanate (FITC) (Alexa Flour 488) at room temperature for 2 h. For general nuclear staining, DAPI (1:200) was added to the final secondary antibody solutions. Finally, sections were mounted and coverslipped with Vectashield mounting medium. 

### 4.10. Quantification Analysis

Immunochemically stained sections were analyzed using Olympus BX-50 fluorescent microscope at 10× and 20× magnifications, captured with digital camera HP Olympus and analyzed by Image J software according to the previous protocol [20,28]. Quantification of GAP-43 positivity was performed on five sagittal sections from rostral and caudal segments of each spinal cord treatment and from sham tissue. Captured digital images were transformed into monochrome 8-bit images and the mean grey level number of black and white pixels within the tissue was determined (value 0–255, when 0 = black pixels, 255 = white pixels). The result yields the mean ratio of black and white pixels expressed by the histogram. The expression of the immunofluorescence (GAP-43) within five sampling fields (perimeter of circle 300 μm) randomly placed above and below the lesion site was evaluated, and a background image of a control section of each image was digitally subtracted. Mean values were calculated as a percentage of GAP-43 axons among SCI + V, SCI + CM four weeks and SCI + CM ten weeks groups and were statistically compared using one-way ANOVA followed by Tukey‘s post hoc test.

### 4.11. Data and Statistical Analysis

Obtained data from tissue analyses and behavioral testing were reported as mean ± SEM. Mean values among different experimental groups were statistically compared by one-way ANOVA and Tukey’s post hoc tests using GraphPad PRISM software. Significant values were denoted with * for *p* < 0.05, ** for *p* < 0.01, and *** for *p* < 0.001.

## 5. Conclusions

Our results confirm the pro-regenerative effects of MScCM on tissue sparing, axonal growth, immunomodulation and final functional outcome. These results suggest that multiple IT delivery of stem cell products may improve behavioral function when the dose and timing are optimized. Taken together, the molecular cocktail found in MScCM is favorable for final neuroregeneration in vivo.
