# Correction: Cizkova, D., et al. Localized Intrathecal Delivery of Mesenchymal Stromal Cells Conditioned Media Improves Functional Recovery in A Rat Model of Contusive Spinal Cord Injury. *Int. J. Mol. Sci.* 2018, *19*, 870

**DOI:** 10.3390/ijms19071942

**Published:** 2018-07-02

**Authors:** Dasa Cizkova, Veronika Cubinkova, Tomas Smolek, Adriana-Natalia Murgoci, Jan Danko, Katarina Vdoviakova, Filip Humenik, Milan Cizek, Jusal Quanico, Isabelle Fournier, Michel Salzet

**Affiliations:** 1 Institute of Neuroimmunology, Slovak Academy of Sciences, Dúbravská cesta 9, 845 10 Bratislava, Slovakia; veronica.cubinkova@savba.sk (V.C.); tomas.smolek@savba.sk (T.S.); adriana.murgoci@savba.sk (A.-N.M.); 2 Department of Anatomy, Histology and Physiology, University of Veterinary Medicine and Pharmacy in Košice, Komenského 73, 041 81 Košice, Slovakia; jan.danko@uvlf.sk (J.D.); katarina.vdoviakova@uvlf.sk (K.V.); filip.humenik@uvlf.sk (F.H.); 3 University Lille, Inserm, U-1192—Laboratoire Protéomique, Réponse Inflammatoire et Spectrométrie de Masse-PRISM, F-59000 Lille, France; Jusal.Quanico@univ-lille1.fr (J.Q.); isabelle.fournier@univ-lille1.fr (I.F.); michel.salzet@univ-lille1.fr (M.S.); 4 Department of Epizootology and Parasitology, University of Veterinary Medicine and Pharmacy in Košice, Komenského 73, 041 81 Košice, Slovakia; milan.cizek@uvlf.sk

The authors wish to make the following correction to their paper [1]: In Figure 1B, we quoted PCDRGs were supplemented with NGF+ but it should have been MScCM. In order to correct this mistake, we have substituted the correct MScCM supplementation in Figure 1B below. This change has no material impact on the rest of the figure, or the legend, results, discussion and conclusions of our paper.

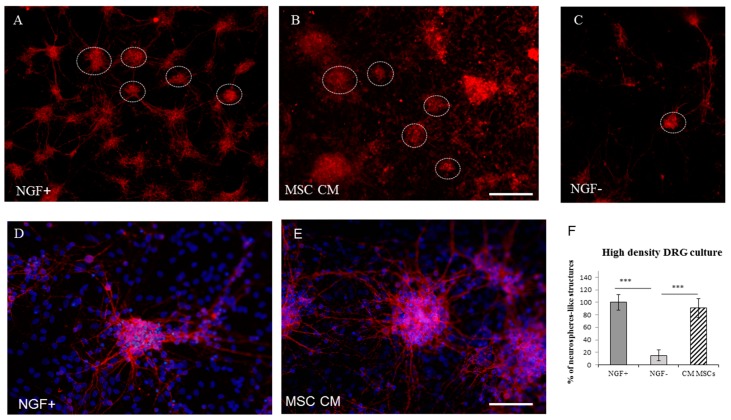



The authors would like to apologize for any inconvenience caused to the readers by these changes. The manuscript will be updated and the original will remain online on the article webpage, with a reference to this Correction.
